# Production of Polyhydroxybutyrate by Genetically Modified *Pseudomonas* sp. phDV1: A Comparative Study of Utilizing Wine Industry Waste as a Carbon Source

**DOI:** 10.3390/microorganisms11061592

**Published:** 2023-06-15

**Authors:** Athina Drakonaki, Eirini Mathioudaki, Ermis Dionysios Geladas, Eleni Konsolaki, Nikolaos Vitsaxakis, Nikos Chaniotakis, Hao Xie, Georgios Tsiotis

**Affiliations:** 1 Department of Chemistry, University of Crete, GR-70013 Voutes, Greece; 2 Max Planck Institute of Biophysics, Max-von-Laue-Straße 3, D-60438 Frankfurt am Main, Germany

**Keywords:** *Pseudomonas* sp. strain phDV1, polyhydroxyalkanoates, phaZ knockout, phaR knockout, PHB

## Abstract

*Pseudomonas* sp. phDV1 is a polyhydroxyalkanoate (PHA) producer. The presence of the endogenous PHA depolymerase (phaZ) responsible for the degradation of the intracellular PHA is one of the main shortages in the bacterial production of PHA. Further, the production of PHA can be affected by the regulatory protein phaR, which is important in accumulating different PHA-associated proteins. PHA depolymerase phaZ and phaR knockout mutants of *Pseudomonas* sp. phDV1 were successfully constructed. We investigate the PHA production from 4.25 mM phenol and grape pomace of the mutants and the wild type. The production was screened by fluorescence microscopy, and the PHA production was quantified by HPLC chromatography. The PHA is composed of Polydroxybutyrate (PHB), as confirmed by ^1^H-nuclear magnetic resonance analysis. The wildtype strain produces approximately 280 μg PHB after 48 h in grape pomace, while the phaZ knockout mutant produces 310 μg PHB after 72 h in the presence of phenol per gram of cells, respectively. The ability of the phaZ mutant to synthesize high levels of PHB in the presence of monocyclic aromatic compounds may open the possibility of reducing the costs of industrial PHB production.

## 1. Introduction

Polyhydroxyalkanoates (PHAs) are biodegradable polyesters synthesized by many bacteria and some archaea [1,2]. These biopolymers are used as an energy and carbon storage material when nutrients such as nitrogen, phosphorus, or oxygen are limited but the carbon sources are in excess [3]. Due to their biodegradability, biocompatibility, and thermoplasticity, PHAs are a promising substitute for synthetic polymers. PHA granules are commonly deposited in the cytoplasm as inclusions for storage [3].

The production of PHAs in response to nutrient starvation depends on the bacterial species and the type of substrate used as a carbon source. While *Bacillus megaterium* was the first bacterium known to produce PHA, the discovery of poly(3-hydroxyoctanoate) in *Pseudomonas oleovorans* showed that other bacteria can synthesize PHA [4]. PHA granules are water-insoluble inclusions that also contain proteins and some lipids [5]. Key enzymes involved in PHA metabolism, such as PHA synthases, PHA depolymerases, regulators, and structural proteins called phasins, are associated with the PHA granule [6]. Regulatory proteins also contribute to the formation of PHA granules by influencing the expression of the structural phasins and themselves [7,8].

The complete genomes of various *Pseudomonas* strains have revealed that these bacteria possess a gene cluster that determines their ability to synthesize and accumulate PHAs [9,10]. PHA-producing Pseudomonads contain the *pha* gene cluster, which encodes the synthase (PhaC) involved in PHA synthesis, a depolymerase (PhaZ) responsible for PHA mobilization, and a putative transcriptional regulator (PhaD) [11,12,13]. In addition, there are other genes that encode phasins and are transcribed divergently from the other *pha* genes, playing regulatory and functional roles [12].

Metabolic engineering has been applied to improve PHA production through various approaches, such as overexpressing the PHA synthesis operon (via plasmid or chromosomal integration) or eliminating the ability to consume PHAs [14]. PHA depolymerases (PhaZ) are responsible for the degradation, and it has been observed that the inactivation of *phaZ* can lead to an increase in PHA accumulation [15]. Deletion of the *phaZ* gene has been shown to improve PHA accumulation in *P. putida* KT2440 using sodium octanoate or lignin as a carbon source [16,17]. The PHA metabolic machinery suggests the existence of intricate regulatory and metabolic networks driven by global and local regulators, which control the pathways involved in carbon and nitrogen assimilation [13]. A repressor protein, PhaR, regulates PHA synthesis by binding to the promoter of phasin (*phaP*) [7]. PhaR regulates a well-organized granule formation process by controlling the expression of PhaP proteins that coat the newly synthesized PHAs [8].

The selection and cost of the carbon source are important parameters to consider for the microbial production of PHA, as they can significantly influence the overall cost-effectiveness of the process [18,19,20,21]. According to the literature, there are a wide variety of microorganisms capable of producing PHA (e.g., *Halomonas*, *Bacillus*, *Ralstonia*, etc.) [22]. *Halomonas* strains have been found to produce PHA by utilizing a wide variety of carbohydrates under high saline conditions [23], whereas *Ralstonia* and *Bacillus* strains require unbalanced or unfavorable growth conditions [24,25]. *Pseudomonas* species have attracted attention for their ability to use a wide range of compounds as carbon sources for PHA production, including various industrial wastes and byproducts [26,27,28]. Recently, the *Pseudomonas* sp. strain phDV1 has been shown to synthesize PHA using monocyclic aromatic compounds as a carbon source [29,30]. The genome of *Pseudomonas* sp. phDV1 contains the key genes involved in PHA biosynthesis and degradation [9]. They can utilize phenol and cresols, up to 600 mg/mL and 200 mg/mL, respectively, as a sole carbon source, converting these toxic compounds into useful products [29,30]. This strain shows potential as a biocatalyst for bioremediation and biosynthesis of biodegradable plastics by removing toxic compounds and producing polyhydroxybutyrates (PHB).

Grapes are a widely cultivated crop used not only for direct consumption but also for the production of various products such as jam, wine vinegar, and grape oil [31]. Wine, particularly in Greece, one of the major wine-producing countries in the EU, is a popular product derived from grapes [32]. However, the processing of grapes into wine generates a variety of wastes, including grape pomace (GP), which is a significant part of the waste from the winery industry. GP from *Vitis vinifera* “Assyrtiko” is an important waste product of the Greek wine industry, and its disposal is a serious issue with negative environmental impacts [33]. GP is rich in bioactive compounds, such as sugars and polyphenols, and its utilization for alternative uses has been of considerable interest [33]. It has been shown that it can be used as a carbon source in various biotechnological processes. While many studies on metabolic engineering in Pseudomonas and other organisms to improve PHA production utilize carbohydrates and oil sources, only a few studies have explored the use of aromatic compounds (GP) as carbon sources [29,30,34,35].

In this study, we aimed to develop an industrially useful strain for PHA production by constructing and characterizing *phaZ* and *phaR* knockout mutants of *Pseudomonas* sp. phDV1. Additionally, we investigated the PHA production characteristics of these mutants and the wild-type strain in a medium containing different carbon sources, including 4.25 mM phenol or grape pomace.

## 2. Materials and Methods

### 2.1. Bacterial Strains, Medium, and Oligonucleotides

*Pseudomonas* sp. phDV1 was used to construct deletion strains of the *phaR* gene (locus_tag, DZC76_00085) and the *phaZ* gene (locus_tag, DZC76_02270), resulting in the ΔphaR and the ΔphaZ strains (Table S1), respectively [9,36,37]. The *Pseudomonas* sp. phDV1 cells were grown in lysogeny broth (LB) medium or M9 minimal medium. Antibiotics were added to final concentrations of 100 μg/mL ampicillin, 50 μg/mL kanamycin, and 170 μg/mL chloramphenicol, if applicable. *Escherichia coli* strain DH5α was used for cloning purposes. DNA sequencing was performed by Eurofins MWG Operon (Ebersberg, Germany). Plasmids and synthetic oligonucleotides (Eurofins MWG Operon) prepared for this study are listed in Tables S2 and S3 in the Supplementary Materials, respectively.

### 2.2. Construction of the ΔphaZ and ΔphaR Strains

The lambda Red recombinase system was used to replace the gene of interest in the *Pseudomonas* sp. phDV1 genome by a kanamycin resistance cassette [38,39]. The gene replacement was carried out using the linearized DNA fragment that contains a kanamycin resistance cassette flanked by regions (~500-bp) upstream and downstream of the target gene. DNA fragments were synthesized by Eurofins MWG Operon and cloned into the pMiniT 2.0 vector using the NEB PCR cloning kit. A helper plasmid, pMK-RedS [40], which contains the genes *araC*, *gam*, *bet,* and *exo*, was electrotransformed into *Pseudomonas* sp. phDV1 cells [41]. The cells containing pMK-RedS were cultured in LB media with 170 μg/mL chloramphenicol at 32 °C and 180 rpm to an optical density at 600 nm (OD600) of 0.5 to 0.6. The expression of proteins Gam, Bet, and Exo was then induced with 0.2% (*w*/*v*) L-arabinose. After 4 h of induction, the *Pseudomonas* sp. phDV1 cells were electrotransformed with approximately 2 μg of linearized DNA and incubated in super-optimal-broth (SOC) medium without antibiotics at 37 °C with shaking (180 rpm) for 2 h. The gene disruption was confirmed by kanamycin resistance selection, PCR, and sequencing (Eurofins MWG Operon, Ebersburg, Germany).

### 2.3. Cultivation of Pseudomonas Strains

GP from *Vitis vinifera* “Assyrtiko” was obtained from the “Diamantakis winery”, an enterprise located in the southwest part of Heraklion, outside Kato Assites village (Crete Island, Greece; latitude 35°12′43 and longitude 24°59′33). The fresh material was collected during the harvest of September 2020 and stored at −20 °C until use. The fresh GP was first dried at 105 °C until a constant weight was achieved and then ground to a fine powder. The extract was obtained by autoclaving the GP powder mixed with deionized water (1.3% *w*/*v*) at 120 °C for 20 min. After this step, any remaining insoluble GP was removed by filtration. *Pseudomonas* sp. phDV1 and its *phaZ* and *phaR* knockout mutants were cultivated in shake flasks in M9 minimal medium using 10 mM succinic acid as the sole carbon source. All bacterial strains were cultivated in M9 medium containing 4.5 mM phenol or GP extract at a final concentration of 1% (*v*/*v*). The cultivation was carried out at 32 °C, and three parallel flasks were arranged for each strain (WT, ΔphaR, and ΔphaZ). Cell growth was monitored by measuring the optical density using a UV2700 UV-vis spectrophotometer at 600 nm (Shimadzu). Cells were collected by centrifugation (6000× *g*, 10 min at 4 °C). The pellet was washed with 0.1 M Tris-HCl pH 7.5 at different time points.

### 2.4. Analytical Methods

Phenol concentration was determined spectrophotometrically at 270 nm using the Thermo Scientific™ Multiskan Sky Microplate Spectrophotometer (Thermo Fischer Scientific, Waltham, MA, USA). The total polyphenolic and carbohydrate content (mg/L) of the GP extract was measured before cultivation (t = 0) and 24 h, 48 h, and 72 h after cultivation (t = 24, t = 48, t = 72, respectively). The Folin-Ciocalteu (FC) assay [42], slightly modified according to Belenioti et al. [43], was used to measure polyphenol concentration. First, two microliters of the sample were pipetted into a well of a 96-well microplate containing 158 μL of deionized water and 10 μL of FC reagent. After 5 min, 30 μL of Na_2_CO_3_ solution were added. After incubation for 30 min at 40 °C, the absorbance of the solution was measured at λ = 765 nm (Multiscan Sky-ThermoScientific, Waltham, MA, USA) against a blank sample consisting of 160 μL of deionized water. The measurements were compared to a standard curve of prepared gallic acid solutions (0–500 mg/L) and expressed as mg of gallic acid equivalents (GAE) per 1 L of GP extract (before and after biodegradation). All measurements were performed in triplicate. The total carbohydrate concentrations were determined according to the thymol-sulfuric acid method [44], as modified by Schulze et al. [45]. A glucose solution (1 mg/mL) was used as a standard.

### 2.5. Analysis of the Polyphenolic Profile of the Culture

The polyphenolic profile of the culture before cultivation (t = 0), 24 h, 48 h, and 72 h after cultivation was analyzed by an HPLC system (Agilent 1260 Infinity II, Santa Clara, CA, USA) with a quaternary pump, diode array detector (DAD), and autosampler according to Belenioti et al. [43]. Separation was carried out on a C-18 column (GraceSmart-ThermoScientific, Waltham, MA, USA; length: 250 mm; i.d.: 4.6 mm). The gradient elution was accomplished by using water (solvent A) and acetonitrile (solvent B), as shown in Table S4. The parameters of the chromatographic process were a 0.8 mL/min flow rate, a 10 μL injection volume, and an analysis time of 23.00 min. The qualification of phenolic compounds was performed using internal standards, and the absorbance was measured at 280 nm.

### 2.6. Nile Red Staining

For sample preparation, 1–2 mL of cells were pelleted by centrifugation (13,000× *g*, 60 s) and resuspended in 50 μL of growth medium. In the reaction tube, add 3 μL of cells to 1 μL of Nile Red solution (250 μg/mL in DMSO). Agarose pads were prepared by pipetting 30 μL of hot (60 °C) 1% (*w*/*v*) agarose solution on a microscope slide. Almost immediately, 4 μL of the stained cell suspension were added to the agarose pad. After a few seconds of drying, the cover slip was placed on the agarose. Cells were observed in a Nikon ECLIPSE E800 microscope (Nikon Instruments Inc., Melville, NY, USA) with an oil-immersed lens (excitation 562/40 nm, emission 594 nm).

### 2.7. Quantification of PHB with HPLC

The quantification of PHB was performed by measuring the crotonic acid absorbance at 215 nm following HPLC. From 20 mL of cell culture, the pellet was harvested by centrifugation (15,317× *g*, 10 min, 4 °C) and washed two times with equal volumes of acetone and ethanol. The conversion of PHB to crotonic acid was completed after digestion of the pellet in 1 mL of concentrated sulfuric acid (Merck, Darmstadt, Germany) for 30 min at 105 °C. After the digestion, the samples were diluted with nanopure H_2_O in a 1:5 volume ratio and filtered using 0.22 μm filters. The filtered samples were analyzed by the Agilent 1260 Infinity II LC System (Agilent, Santa Clara, CA, USA). The samples were loaded on the reversed-phase column InfinityLab Poroshell 120 EC-C18 (4 μm pore size, 4.6 × 150 mm, Agilent, Santa Clara, USA) and eluted with 0.5 mL/min, 85% phosphoric acid solution (0.1 M, Honeywell, NC, USA), and 15% (*v*/*v*) acetonitrile (Fisher Scientific, Portsmouth, NH, USA), at 30 °C. Furthermore, the crotonic acid in the samples was detected by a diode array detector at 215 nm and quantified based on a standard curve.

### 2.8. Isolation of PHB Granules

Cells were disrupted by sonication with an Ultrasonicator Processor UP200s (Hielscher, Bonn, Germany) with 30% amplitude and one cycle (10 times, 15 s with 45 s intervals), taking into consideration that the temperature should be below 10 °C. Approximately 1.5 mL of broken cell suspension were layered on a discontinuous sucrose gradient. The gradient consisted of 3 mL of 2.0, 1.67, and 1.33 M sucrose in 0.1 M Tris-HCl, pH 7.5. After ultracentrifugation at 210,000× *g* for 3 h at 4 °C, the white layers corresponding to the PHB granules were collected and washed to remove the sucrose with 0.1 M Tris-HCl pH 7.5. The washed granules were resuspended in 0.1 M Tris-HCl, pH 7.5, and stored at −20 °C for further analysis.

### 2.9. NMR Spectroscopy

The isolated PHB samples or PHB granules were dissolved in 600μL of deuterated chloroform, CDCl_3_, and transferred into 5 mm NMR tubes (Deutero GmbH, Kastellaun, Germany) after they had been dissolved via sonication. Experiments were performed in a Bruker DPX-300 spectrometer (Bruker, Billerica, MA, USA) at 298 K using standard Bruker pulse program libraries. Spectral processing and analysis were performed using TopSpin 4.0 (Bruker, Billerica, MA, USA) software. All chemical shifts reported are referenced to the residual chloroform peak (*δ* 7.26 ppm).

### 2.10. Data Analysis

All the data presented in this study was visualized using Origin Pro 9, using the means and standard deviations (SD) as error bars. Note that each mean and SD were the result of at least three different biological replicates.

## 3. Results and Discussion

### 3.1. Construction of ΔPhaZ and ΔPhaR Mutants of Pseudomonas sp. phDV1

**Figure 1:**
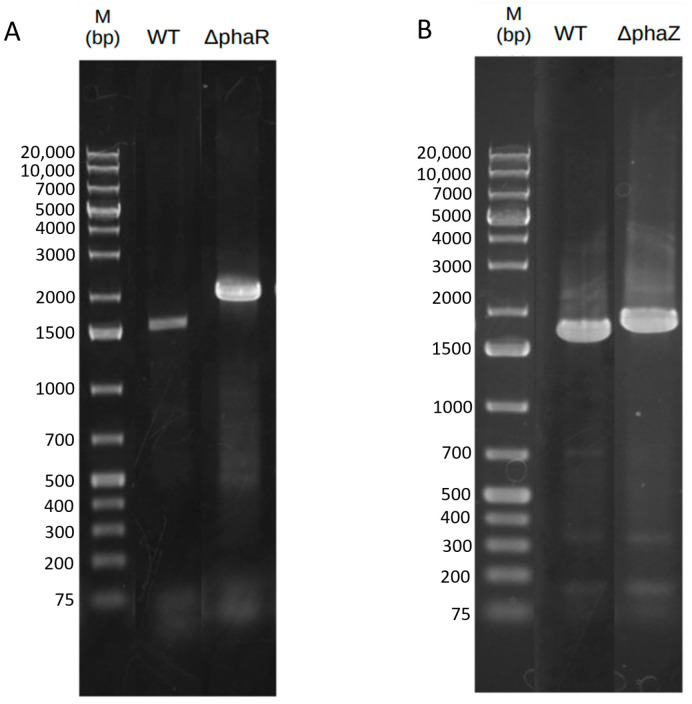
PCR electrophoresis of the phaR (**A**) and phaZ (**B**) knockout mutants. Lane M: marker; Lanes phaR and phaZ: negative control using *Pseudomonas* sp. phDV1 genomic DNA as the template; Lanes ΔphaR and ΔphaZ genomic DNA of single colonies selected on the screening plates were ΔphaR and ΔphaZ knockout mutants.

To enhance PHB accumulation, *Pseudomonas* sp. phDV1 was genetically modified to eliminate PHB depolymerization or disrupt its regulation. The *phaZ* gene, which codes for the depolymerase of PHB (284 residues), was located between the class II poly(R) polymerase (DZC76_RS02265) gene and a pseudogene (DZC76_RS02275) (Figure S1), while the *phaR* gene, which encodes a repressor protein (174 residues) that affects the production of PHB, was located between a phasin family protein gene (DZC76_RS00080) and gene corresponding to a hypothetical protein (DZC76_RS00090) (Figure S2) [7,9]. Both ΔphaZ and ΔphaR mutants were successfully generated by disrupting the respective genes in the genome using a kanamycin resistance cassette. PCR analyses were carried out to confirm the insertion of the kanamycin resistance cassette and the deletion of *phaZ* or *phaR*, using genomic DNA extracted from the wild-type and the corresponding knockout strains as templates (Figure 1).

In the case of the ΔphaR knockout strain, primers phaR-F and phaR-R were used, whereas in the case of the ΔphaZ knockout strain, primers phaZ-F and phaZ-R were used (Table S3). The disrupted genes showed larger PCR products than the wild-type, with the ΔphaR knockout strain producing a PCR product of 2177 bp compared to the wild-type of 1744 bp and the ΔphaZ knockout strain producing a PCR product of 1996 bp compared to the wild-type of 1949 bp.

### 3.2. Pseudomonas sp. phDV1 Cell Growth in Grape Pomace Extract

The main objective of the present study is to design a bioprocess for the production of PHAs with high yields using the appropriate mutant of *Pseudomonas* and cheap, sustainable carbon sources for the initial growth phase. GP from *Vitis vinifera* “Assyrtiko” was chosen for this purpose because it is rich in sugars and polyphenols, as “Assyrtiko” grapes are used for the production of white wine. During the wine-making process, pressing is performed before alcoholic fermentation, which results in pomace naturally enriched with sugars such as glucose and fructose [46]. Additionally, significant amounts of polyphenols remain in the pomace, making it an ideal carbon source for bacterial growth.

**Figure 2:**
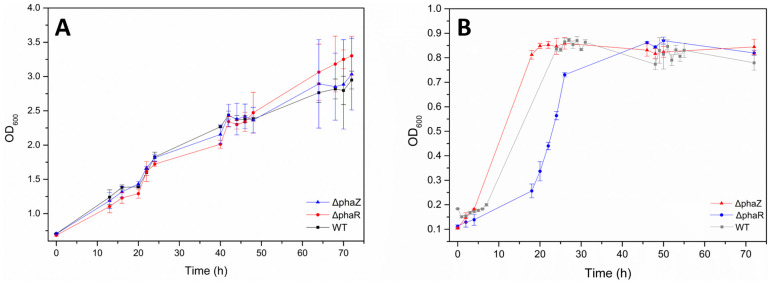
Growth of WT and ΔphaR and ΔphaZ *Pseudomonas* sp. phDV1 strains in minimal salt medium supplied with 1% GP extracts (**A**) and 4.5 mM phenol (**B**).

Initially, we tested the growth of *Pseudomonas* sp. phDV1 (wildtype, ΔphaZ, and ΔphaR) in M9 minimal medium supplemented with 1% (*v*/*v*) of GP extract. Cell growth was monitored by measuring OD at 600 nm from t = 0 to t = 72 (total time points: t = 0, t = 24, t = 48, and t = 72). According to the cultivation curves (Figure 2A), wild-type, ΔphaZ and ΔphaR cells demonstrated high rates in the presence of GP extract and reached higher ODs than when grown in M9 medium supplemented with 4.5 mM phenol as a carbon source (Figure 2B). Indicatively, the dry cell mass corresponding to an OD of 0.89 is 14.56 mg/mL for WT cultivated in GR extract.

Therefore, we can conclude that *Pseudomonas* sp. phDV1 is capable of growing in an environment containing GP from *Vitis vinifera* “Assyrtiko” at a final concentration of 1% (*w*/*v*). Compared to growth in the presence of phenol alone, we observed a delay in growth, which may be due to the longer adaptation time required for the GP extract. According to a one-phase exponential growth function with a time constant parameter, we estimated doubling times in the range of 0.2–13 h for phenol and 28–83 h for the GP extracts. Bacterial cells need to promptly adapt their growth to changing nutritional conditions to preserve population fitness. In order to meet the metabolic demand, bacteria must change their gene expression pattern and metabolome during the nutrition transition, upregulating the expression of necessary proteins and downregulating the expression of unnecessary ones [47]. The longer doubling time observed for all *Pseudomonas* strains in the GP extracts indicates a longer adaptation time for this carbon source.

### 3.3. Biodegradation of Sugars and Polyphenols

As it was described in the M&M section, GP extract was obtained by autoclaving a 1.3% (*w*/*v*) dried GP-water mixture for 20 min. After 20 min of extraction at 120 °C, the liquid fraction was diluted in M9 medium at a final concentration of 1% (*v*/*v*). This mixture was analyzed before being used as a medium for *Pseudomonas* sp. phDV1 growth, and it was found to be rich in sugars and polyphenolic compounds. Specifically, it contained 364 mg/L of total polyphenols as well as 19 g/L of total sugars. The total polyphenolic content as well as the sugars of the cultures were measured at different time points to monitor the ability of *Pseudomonas* to degrade those compounds. According to the literature, glucose and fructose are the major constituents of GP, comprising over 78% of the total soluble sugars [48]. In addition, low levels of sucrose are detected in GP samples, corresponding to the low sucrose content in grapes and the propensity of sucrose to be hydrolyzed into glucose and fructose [49]. Furthermore, GP is a substitute for polyphenols, with catechin and epicatechin being the most abundant compounds [43].

**Figure 3:**
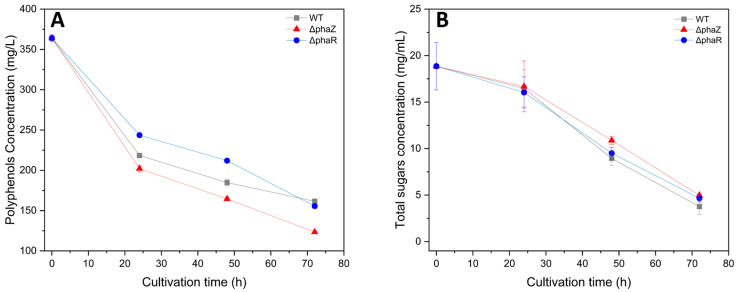
Consumption profiles of polyphenols of wildtype and ΔphaR and ΔphaZ *Pseudomonas* sp. phDV1 strains (**A**). Consumption profiles of sugars of wildtype and ΔphaR and ΔphaZ *Pseudomonas* sp. phDV1 strains (**B**).

Initial experiments indicated that the wild-type strain primarily metabolized glucose when cultivated in M9 medium containing glucose and a standard polyphenol (gallic acid) as carbon sources. As shown in Figure 3, the bacterium was able to use both polyphenols and sugars as carbon sources. Specifically, the wildtype strain managed to reduce the total polyphenolic content by 55.6%, while the ΔphaZ and ΔphaR strains decreased it by 66% and 57.3%, respectively. On the other hand, WT decreased the total sugars by 80%, while the ΔphaZ and ΔphaR strains decreased them by 74% and 75.6%, respectively. These results suggest that *Pseudomonas* sp. phDV1 can use both polyphenols and sugars as carbon sources, with a slight preference for polyphenolic compounds. The strain has been reported to have the ability to degrade aromatic compounds [9,29,37,50] and can utilize glucose as an energy source [51]. The delayed use of carbohydrates as a carbon source may be due to their slower transport into the cell compared to hydrophobic aromatic compounds.

**Figure 4:**
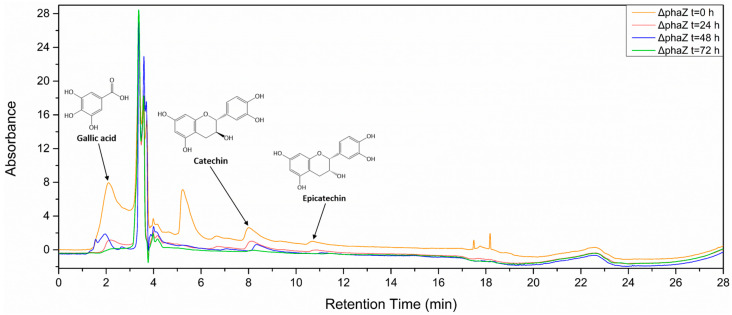
Chromatogram of GP extract at different points of cultivation (0, 24, 48, and 72 h).

In addition, we analyzed the polyphenolic profile of the culture at four different time points (t = 0, t = 24, t = 48, and t = 70 h after cultivation) using an HPLC system. Figure 4 shows the chromatographs of GP extract in the culture of the ΔphaZ mutant. The identification of polyphenols was performed by comparing them to internal standards. The results indicate that the most abundant polyphenols in *Vitis vinifera* “Assystiko” are gallic acid, catechin, and epicatechin, which is consistent with the findings of Belenioti et al. [43]. Moreover, Figure 4 illustrates that all identified polyphenols decreased with the increase in cultivation time, suggesting that the ΔphaZ mutant is capable of degrading gallic acid, catechin, and epicatechin and using these polyphenols as a carbon source. The WT *Pseudomonas* sp. phDV1 and ΔphaR mutants showed similar results regarding their biodegradation abilities.

According to previous studies, several pathogens, such as *Serratia marcescens*, *Escherichia coli*, *Proteus mirabilis*, *Bacillus cereus*, *Pseudomonas aeruginosa*, and *Candida albicans*, have been found to be resistant to catechins, particularly those found in tea [52,53]. However, in contrast to these findings, *Pseudomonas* sp. phDV1 in our study is tolerant to catechin, epicatechin, and gallic acid, achieving an OD of 1.61 (wild-type), 1.65 (ΔphaR), and 1.8 (ΔphaZ) 72 h after cultivation. This discovery sheds light on how polyphenols can affect microbial growth, as most studies on the interactions between polyphenolic compounds and microorganisms have focused on their antimicrobial effects against pathogens [52,54,55], with only a few investigating their positive effects on bacterial growth [53,56]. For instance, *Schenedesmus obliquus*, a green alga, has been shown to biodegrade phenolic substances [57,58]. *Chlamydomonas rheinhardii* has been found to successfully degrade catechin and epicatechin in *Vitis vinifera* “Assyrtiko” extract. Furthermore, it has been discovered that choosing the proper cultivation conditions is critical for increasing polyphenolic biodegradation.

### 3.4. PHB Production from Grape

**Figure 5:**
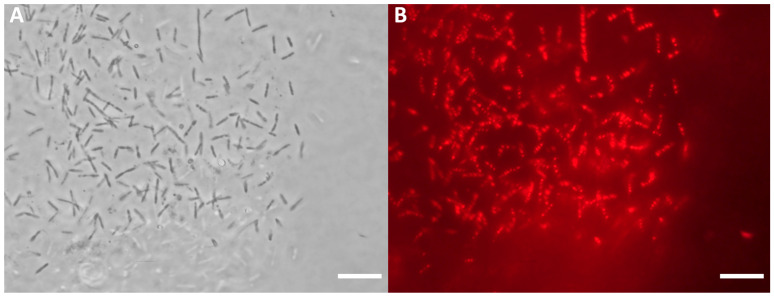
Accumulation of PHB in the *Pseudomonas* sp. phDV1 ΔphaZ strain. (**A**) Optical microscopy of *Pseudomonas* sp. phDV1 ΔphaZ strain (**B**) *Pseudomonas* sp. phDV1 ΔphaZ strain expressed fluorescence when stained with Nile Red. The cells were grown in 500 mL of medium containing 1% GP waste after 48 h. The PHB production is seen as a red fluorescence. The scale bar is 10 μm.

The ability of *Pseudomonas* sp. phDV1 to produce PHB has been previously confirmed by Kanavaki et al. [30]. In this study, we investigate the ability of different mutants of this bacterium to utilize different waste materials as carbon sources in order to produce PHB. The GP extract from the Greek white grape variety “Assyrtiko”, supplemented by the components of the cultivation medium, was tested. Figure 5 shows a microscope image of the ΔphaZ cells stained with Nile Red 48 h after cultivation, revealing the production of PHB within the cells. The optical microscopy findings indicate that the ΔphaZ mutant was able to produce the highest concentration of PHB 48 h after cultivation (Supplementary Materials, Figure S3). Additionally, ΔphaZ produced the highest yield of PHB among the three strains (Supplementary Materials Figure S3). The PHB content in the dry cell mass of the ΔphaZ strain was found to be 16% after 48 h of cultivation.

**Figure 6:**
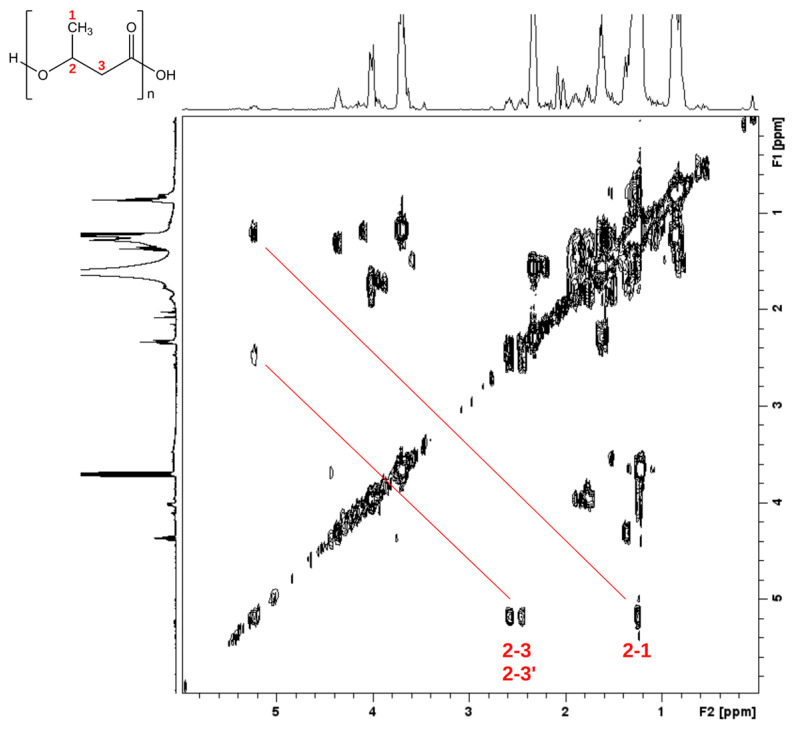
Homonuclear ^1^H-^1^H 2D gCOSY NMR spectra of isolated PHB granules produced by *Pseudomonas* sp. phDV1 grown in M9 minimal media supplemented with 4.5 mM phenol as the sole carbon source. The spectra were recorded in CDCl_3_ solution at 500.13 MHz.

**Figure 7:**
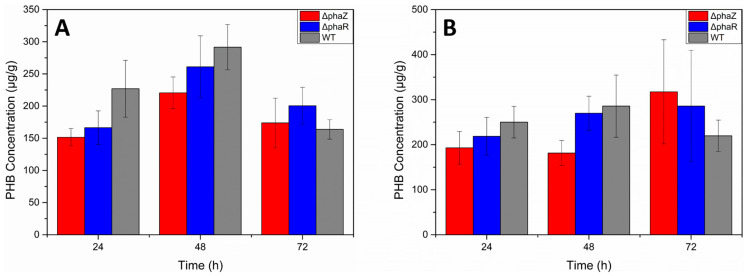
Effect of different carbon sources in correlation with the dry cell weight in PHB production by *Pseudomonas* sp. phDV1 WT, phaR, and phaZ strains (**A**) in M9 medium containing 1% GP waste (**B**) in M9 medium containing 4.5 mM phenol.

^1^H-NMR analysis was conducted to characterize the produced polymers. PHB granules were isolated from broken cells after sucrose density separation. The ^1^H NMR spectrum of the PHA granules in CDCl_3_ solution (top projection in Figure 6, Supplementary Materials Figure S4) showed a signal at δ 5.26, at δ 2.61, and at δ 1.47 with integral ratios of 1:2:3, respectively. These ^1^H NMR spectral data (chemical shift and scalar couplings) are identical with those reported for PHB isolated from *Pseudomonas* sp. phDV1 using phenol as a carbon source [30]. To overcome signals arising from impurities in the sample, we recorded a 2D heteronuclear ^1^H-^1^H gCOSY NMR experiment of the isolated PHA material, depicted in Figure 6, which shows correlations between neighboring protons connected via scalar *J* couplings. In the gCOSY 2D NMR spectrum, the methine proton (2) is clearly connected via *J* coupling not only with the two methylene protons (3, 3′), as expected, but also with a signal at δ 1.27, which coincides exactly with the chemical shift reported for the methyl protons (1) of PHB [30]. In order to confirm all these qualitative findings, the actual concentration of PHB produced by each mutant at four different time points (t = 0, t = 24, t = 48, t = 72) was quantified using HPLC. The highest PHB yields were achieved for wild type 48 h after cultivation when GP was used as the sole carbon source (Figure 7). In contrast, in the presence of 4.5 mM, the highest PHB yields were achieved for ΔphaZ 72 h after cultivation.

## 4. Conclusions

*Pseudomonas* sp. phDV1 was shown to be capable of producing PHB using grape pomace extract as a carbon source, with the ΔphaZ mutant yielding the highest amounts of the biopolymer after 48 h of cultivation compared to WT and ΔphaR strains. Nonetheless, utilizing waste sources such as grape pomace extracts to produce PHB with *Pseudomonas* sp. phDV1 is a promising alternative to traditional PHB production methods, as it increases the competitiveness of the production process by using waste as an input source.

## Data Availability

All data are available in the manuscript.

## Supplementary Materials

The following supporting information can be downloaded at: https://www.mdpi.com/article/10.3390/microorganisms11061592/s1. Figure S1: Schematic representation of the generation of ΔphaR knockout mutant; Figure S2: Schematic representation of the generation of ΔphaZ knockout mutant; Figure S3: Accumulation of PHB in the *Pseudomonas* sp. phDV1 strain and knockout mutants. Optical and fluorescence microscopy. (A) Upper-line *Pseudomonas* sp. phDV1 grown in GP extracts. Middle-line *Pseudomonas* sp. phDV1 ΔphaZ strain grown in GP extracts. Lower-line *Pseudomonas* sp. phDV1 ΔphaR strain grown in GP extracts. (B) Upper-line *Pseudomonas* sp. phDV1 grown in 4.5 mM phenol. Middle-line *Pseudomonas* sp. phDV1 ΔphaZ strain grown in in 4.5 mM phenol. Lower-line *Pseudomonas* sp. phDV1 ΔphaR strain grown in in 4.5 mM phenol.; Figure S4: ^1^NMR spectra of the isolated PHB granules from the *Pseudomonas* sp. phDV1 ΔphaZ strain grown with 1% GP extracts (A) and 4.5 mM phenol (B). Table S1: *Pseudomonas* sp. phDV1 strains used in this study; Table S2: Plasmids used in this study; Table S3: Oligonucleotides used in this study; Table S4: The gradient of elution solvent.
