# Supplementary material for: Production of Polyhydroxybutyrate by Genetically Modified Pseudomonas sp. phDV1: A Comparative Study of Utilizing Wine Industry Waste as a Carbon Source
Source: Microorganisms. 2023 Jun 15;11(6):1592. doi: 10.3390/microorganisms11061592 (PMC10304062; doi:10.3390/microorganisms11061592)
Supplement: Supplementary file 1 [file microorganisms-11-01592-s001.zip › microorganisms-2333452-supplementary.pdf]

Supplementary Materials

Table S1: *Pseudomonas* sp. phDV1 strains used in this study.

**Strains**	**Features**	**Reference**
Pseudomonas sp. phDV1	Wild-type	[9,32,33]
ΔphaZ	*Pseudomonas* sp. phDV1 Δ*phaZ*::Kan^r	This work
ΔphaR	Pseudomonas sp. phDV1 Δ*phaR*::Kan^r	This work

Table S2: Plasmids used in this study.

**Plasmids**	**Features**	**Reference**
pMiniT 2.0	Blunt cloning plasmid	NEB
pMK-RedS	pBBR1MCS derivative; *gam*, *bet*, and *exo* from pUCP18-RedS cloned into pBBR1MCS, Cam^r	[36]
pMini-ΔPhaZ	pMiniT derivative, a kanamycin resistance cassette flanked by regions (~500-bp) upstream and downstream of *phaZ*	This work
pMini-ΔPhaR	pMiniT derivative, a kanamycin resistance cassette flanked by regions (~500-bp) upstream and downstream of *phaR*	This work

Table S3: Oligonucleotides used in this study.

**Oligonucleotides**	**Sequence (5'→ 3')**	**Description**
phaR-F	CACGACCTGCAGGGCAGCAAC	amplification of the linearized DNA fragment for ΔphaR
phaR-R	TGCGTGACCCGCAGGCCTTC	
phaZ-F	ACGCCATGGGACTCCTGCTAC	amplification of the linearized DNA fragment for ΔphaZ
phaZ-R	GAGGTGCCAGGTCGGATCAGC	
pMini-F	ACCTGCCAACCAAAGCGAGAAC	sequencing primers for pMiniT plasmid derivatives
pMini-R	TCAGGGTTATTGTCTCATGAGCG	

Table S4 The gradient of elution solvents.

**No**	**Time (min)**	**Solvent A**	**Solvent B**
1	0.00	90.0	10.0
2	10.00	85.0	15.0
3	12.00	80.0	20.0
4	14.00	60.0	40.0
5	16.00	40.0	60.0
6	18.00	5.0	95.0
7	20.00	40.0	60.0

8	22.00	75.0	25.0
9	23.00	90.0	10.0

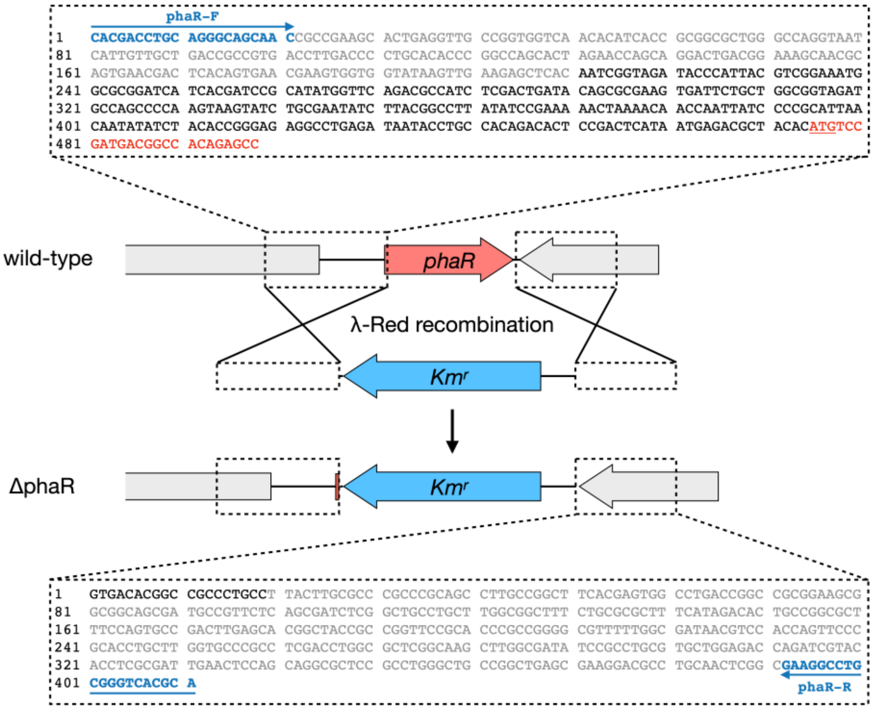

FigureS1. Schematic representation of the generation of ΔphaR knockout mutants.

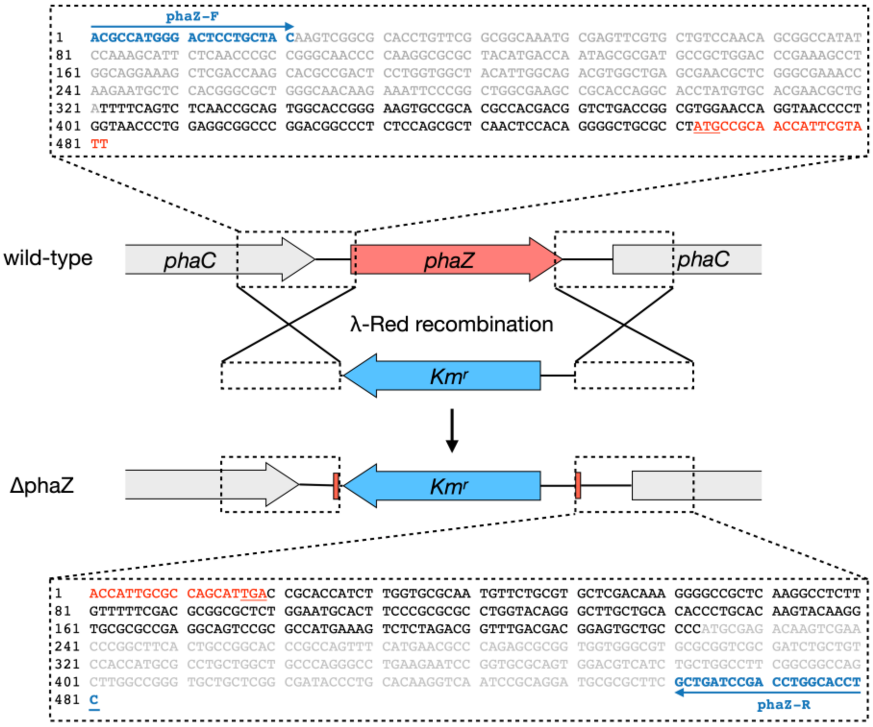

Figure S2. Schematic representation of the generation of ΔphaZ knockout mutants.

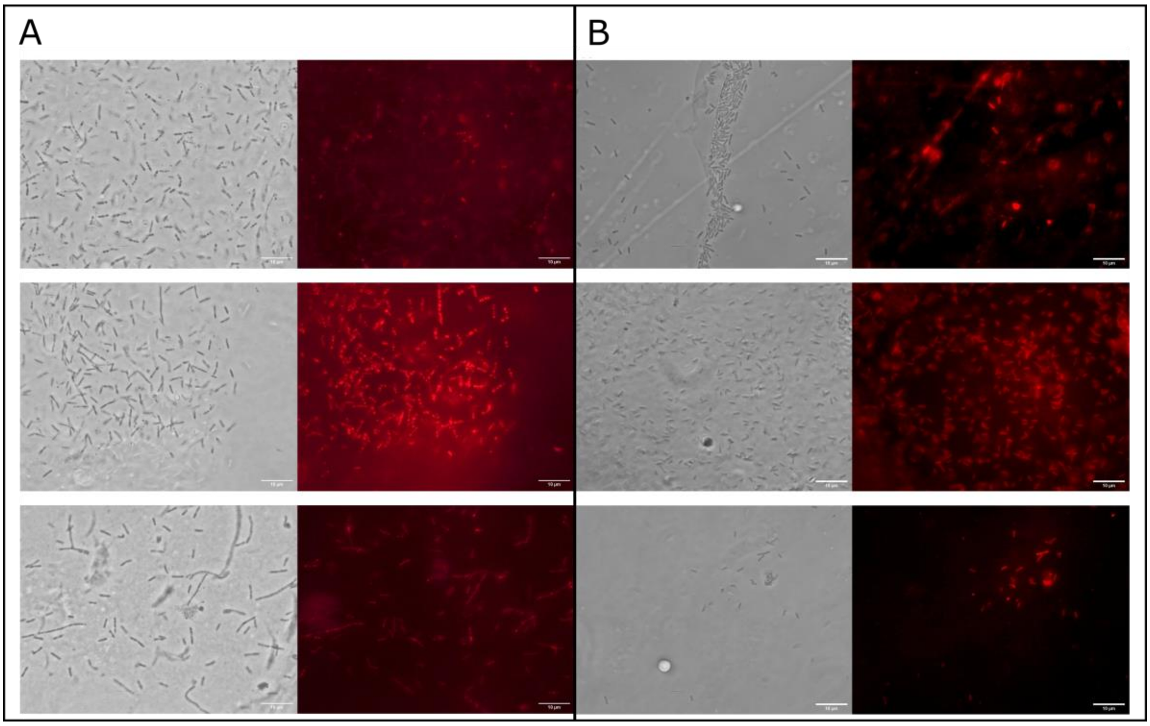

Figure S3. Accumulation of PHB in *Pseudomonas* sp. phDV1 strain and knockout mutants. Optical and fluorescence microscopy. (A) Upper-line *Pseudomonas* sp. phDV1 grown in GP extracts. Middle-line *Pseudomonas* sp. phDV1 ΔphaZ strain grown in GP extracts. Lower-line *Pseudomonas* sp. phDV1 ΔphaR strain grown in GP extracts. (B) Upper-line *Pseudomonas* sp. phDV1 grown in 4.5 mM phenol. Middle-line *Pseudomonas* sp. phDV1 ΔphaZ strain grown in 4.5 mM phenol. Lower-line *Pseudomonas* sp. phDV1 ΔphaR strain grown in 4.5 mM phenol.

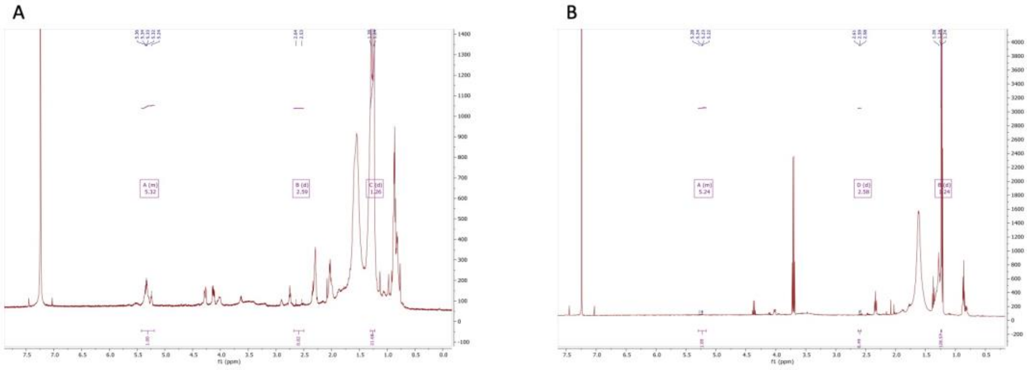

FigureS4. ^{1}NMR spectra of the isolated PHB granules from the *Pseudomonas* sp. phDV1 ΔphaZ strain grown with 1% GP extracts (A) and 4.5 mM phenol (B).
